# Relationship between low mood and micro-expression processing: evidence of negative bias in interpreting fleeting facial expressions

**DOI:** 10.1098/rsos.231944

**Published:** 2024-07-31

**Authors:** Kasia Wezowski, Ian S. Penton-Voak

**Affiliations:** ^1^ School of Psychological Science, University of Bristol, 12a Priory Road, Bristol BS8 1TU, UK; ^2^ National Institute for Health Research Bristol Biomedical Research Centre, University Hospitals Bristol NHS Foundation Trust and University of Bristol, Bristol, UK

**Keywords:** depression, facial expression, micro-expression recognition

## Abstract

Depression affects the recognition of emotion in facial expressions by reducing the detection accuracy and adding a bias towards negativity. However, no study has examined associations between depression and the recognition of microfacial expressions (fleeting facial cues of emotions in people’s faces). Thus, we investigated associations between low mood and micro-expression processing using video stimuli of micro-expressions. We examined whether (i) individuals with low mood had trouble recognizing emotions, (ii) were more likely to perceive happy facial expressions as neutral and neutral facial expressions as sad, and (iii) recognized sad emotional expressions better than control subjects (*n* = 349). We found that participants with low mood showed poorer performance when judging emotions in faces (*p* = 0.03). Furthermore, there was a specific deficit among them in recognizing happiness. Lastly, participants with low moods were more likely to perceive neutral faces as sad (*p* = 0.042). However, no evidence was found that individuals with low moods confused happy faces as neutral or were better than the control group at recognizing sad faces. Our results show that mood affects the perception of emotions in facial expressions, which has the potential to negatively affect interpersonal interactions and ultimately quality of life.

## 1. Introduction

Depression is a leading cause of disability worldwide, affecting approximately 280 million individuals globally, estimated based on the Global Health Data Exchange data source query tool (https://vizhub.healthdata.org/gbd-results). It is one of the most prevalent affective disorders and is distinguished by enduring feelings of sadness, anhedonia and irritability [1,2]. The World Health Organization (WHO) ranks depression as the fourth leading contributor to the global disease burden owing to the effects of depression on productivity and its significant impact on affected individuals, their families and society at large [3].

Aside from the effects on the cognitive and emotional aspects of one’s life, depression also impairs social functioning [4]. The prevalence of deficits in social skills in people suffering from major depressive disorder (MDD) is approximately 43.3%, considerably influencing their quality of life [5]. An important aspect of social functioning is understanding social cues correctly, which is something that people with depression may struggle with [6–8], due to cognitive biases associated with the disease. Research into interpretation bias shows that individuals with MDD have a tendency to interpret ambiguous situations as negative, especially when self-referential stimuli are involved [9]. Research has shown that various types of stimuli may be affected by this bias [10], including facial expressions.

Research on the effects of depression on facial expression recognition showed two main findings. One is that there is an overall negative effect on the accuracy of facial expression recognition, which has been demonstrated in a meta-analysis as robust and stronger than a similar effect associated with anxiety [11]. The deficit in facial expression recognition has also been recognized in more recent research [12–14] and is generally attributed to a deficit in cognitive processing associated with depression. The second effect is that there is a bias in expression recognition towards sadness—depressed people tend to see happy faces as neutral and neutral faces as sad [13–17]. A longitudinal study investigated whether this effect persists after a six-month treatment with antidepressants. The authors found that, even though scores on depression were lowered, faster recognition of sad faces (in comparison with happy ones) persisted [18]. It has also been determined that depression has an effect on the recognition of other emotions, such as disgust [16], fear [14,19] and surprise [15].

While the available research in the field shows that there is a relationship between micro-facial expression emotion recognition and depression, there is an important gap insofar as the understanding of micro-expression processing and its relationship to depression. Micro-expressions refer to the spontaneous facial expressions of seven emotions—anger, contempt, disgust, fear, happiness, sadness and surprise—that are elicited in less than half a second [20–23]. Micro-expressions are involuntary and difficult to hide, and thus appear even when we are trying to deceive others [24], and could be detectable even if a person is trying not to convey their honest emotions. As such, they are very important in social interactions [25], and it is very important to determine how they are affected by other variables. While most of the previously mentioned studies [11–17] did utilize short exposition times (100–500 ms) when presenting emotions, they did so using static images, thus reducing the ecological validity of the studies. In real-life situations, micro-expressions have a specific way of appearing and disappearing from a person’s face, which is only partially reproduced by showing a facial expression for a short time. Furthermore, micro-expressions are very subtle and may involve the movement of only a limited number of muscles to a limited degree. This is in contrast with macro-expressions, which usually last longer and can be much more pronounced [26]. Therefore, the real-life experience of seeing micro-expressions can hardly be captured in a static form. Given that micro-expressions are often indicative of honest, possibly hidden emotions, their detection can be of great value in social interactions [24]. Hence, the relationship between mental health disorders, including depression and its subclinical forms (low mood) can be very important for the understanding of the interplay among cognition, social skills and mental health.

Thus, to elucidate the relationship between depression and micro-expression processing, we sought to examine the differences in micro-expression recognition between people who indicated low mood and control participants who did not report it. Since it was not feasible to clinically determine whether study participants were suffering from depression, we used depression symptoms assessed by the Beck Depression Inventory-II (BDI-II) [27] as a proxy for depression and interpreted the results as indicators of mood. The study tested the following hypotheses:

1. People with higher BDI-II scores will show impaired micro-expression recognition compared with people with lower BDI-II scores.
2. People with higher BDI-II scores will more commonly misattribute happy faces as neutral compared with people with lower BDI-II scores.
3. People with higher BDI-II scores will more commonly misattribute neutral faces as sad compared with people with lower BDI-II scores.
4. People with higher BDI-II scores will exhibit improved recognition of sad faces compared with people with lower BDI-II scores.

As this was the first study examining the relationship between low mood and micro-expression processing, the participants were tested on the recognition of emotions other than happiness and sadness, but these analyses were purely exploratory and there were no hypotheses.

## 2. Material and methods

### 2.1. Study design

This study used an observational design to investigate the differences in facial expression recognition, as assessed by the micro-expression training videos test (METV) [28], between people with low mood (assessed by BDI-II scores) and participants with no mood issues. The primary outcome was the overall score on the METV test. Low mood and control groups were created by separating the sample into two halves based on the BDI-II scores. Exploratory analyses were also conducted on the upper and lower 25% of the sample grouped into low BDI scores (very low mood) and high BDI scores (high mood) groups. We also collected the following demographic data: age in years, gender (male, female and other), highest level of education and occupational status (e.g. upper management, junior management, student, consultant, etc.).

### 2.2. Participants and recruitment

Recruitment took place via Prolific. Participants who completed the study were reimbursed £4. The Prolific platform encourages registered participants to complete a variety of questionnaires to determine likely eligibility for future research studies. For this study, we recruited two groups of participants on the basis of self-reported depression (or its absence) in these screening questionnaires. Since the screening questions were filled out at the point of signing up to Prolific, this determination was done at unknown and various times before the doing of the present study. Therefore, the purpose of using this question during screening was to achieve heterogeneity in terms of levels of depression among the participants. However, study groups were created by splitting the participants into two equally sized groups based on the median BDI-II score, a more objective measure of the degree of depressive symptomatology experienced by participants.

#### 2.2.1. Inclusion criteria

Participants in both groups who were aged 18 and over and were native speakers or equivalently fluent in English were included in the study.

#### 2.2.2. Exclusion criteria

The initial research proposal included the exclusion criterion of antidepressant use. However, the Prolific platform does not provide the capability to exclude participants based on antidepressant usage. Instead, participants were queried regarding their antidepressant usage as part of the survey. A substantial proportion of participants in the sample (15%) reported antidepressant usage, rendering their exclusion unfeasible without significantly diminishing the study’s statistical power. Moreover, given the significant representation of antidepressant users within the sample, we deemed it important to include these participants in the analysis, as they constitute a notable segment of the target population. Finally, in order to check for the effects of this group of participants, sensitivity analysis was conducted by excluding these participants from the sample and re-doing the analyses.

#### 2.2.3. Sample size determination and rationale

There are multiple effect sizes concerning negative biases in emotional face judgements found in the literature. When it comes to biases (recognizing one emotion as another), effect sizes have been around *d* = 0.4 [16,18], while overall emotion expression effect sizes have been higher (*d* = −0.58), and sad face detection effects even higher (*d* = 0.7) [11,18], although other meta-analyses have reported much lower effect sizes (e.g. Dalili *et al*. [29] reported *g* = −0.16). As the present study investigated all three phenomena, we opted for expecting a medium effect size found in previous literature (*d* = 0.4).

A sample size calculation performed in G*Power software [30] indicated that a sample size of 328 was required to achieve the *d* = 0.4 effect size at 0.95 power (and an effect size of *d* = 0.3 at approximately 80% power) using a *t*-test for independent samples, the most basic comparison of the studied groups. However, an initial sample size of 350 was used (175 per group) to ensure a sufficient number of participants after outliers and non-completers were removed from the sample.

#### 2.2.4. Withdrawal of participants

Participants were informed that they could withdraw from the study at any time by leaving the study webpage. Participants who opted out before completing the survey did not receive a reimbursement.

### 2.3. Measures and materials

#### 2.3.1. The Beck Depression Inventory-II

The BDI-II is used to measure depressive symptoms and was used in the present study as a measure of low mood. It consists of 21 items and asks the participant to describe themselves based on their experience in the past two weeks. Specifically, the BDI-II assesses how often participants experience certain depressive phenomena, such as agitation or concentration difficulty [27]. Items were scored on a 4-point Likert scale ranging from 0 to 3. Scores are calculated by adding up all answers, where higher scores indicate severe depression. The scale has excellent reliability (average Cronbach’s α in a meta-study 0.9) and validity [31].

#### 2.3.2. Micro-expressions training videos

METVs were used to measure the recognition of facial expressions. METVs [28] are based on the facial action coding system (FACS) rules developed by Paul Ekman and Wallace V. Freisen [32]. However, contrary to other micro-expression recognition programmes such as Paul Ekman’s METT [33], the METV programme does not use pictures of faces (static stimuli); instead, it uses videos showing facial expressions in a real interaction (i.e. videos of people having a conversation, speaking or listening). For the purpose of the study, only 20 videos were randomly selected from the total of 300 videos, so that there was one video for each aspect of the facial expression of an emotion. The selection of the videos for the METV test is based on the theory of facial muscle movements. Some micro-expressions, such as anger or sadness, can be shown on the face in a number of different ways. Surprise and fear have fewer facial muscle movement variations, disgust and contempt even fewer and happiness is primarily determined by the position of the lips [32,34]. The METV test consists of the following 20 videos: four micro-expressions of anger, four of sadness, three of fear, three of surprise, two of disgust, two of contempt, one of happiness and one neutral face. Each video consists of one white male or one white female face looking straight at the camera with only one variation of a micro-expression at normal speed. The order of the videos is randomized, and an equal number of videos featuring both genders are shown (10 each). The presentation of either a male or female face for a specific emotional expression is randomized for participants. The duration of the chosen micro-expressions was 0.5 s or shorter. Micro-expressions shown on faces in the videos were triggered naturally and complied with the FACS coding rules [32]. They were coded by two independent certified FACS coders, guaranteeing FACS compliance. The METV research test takes approximately 10 min to complete. The METV test is a forced choice test of one from eight possible answers (representing each emotional category that is displayed during the test). The METV test allows participants three attempts to provide an answer if their response is not correct. In the present study, the score was calculated as the proportion of correct answers given on the first attempt.

### 2.4. Procedure

The study involved a single online session lasting approximately 20 min, with activities occurring in the following order: demographic questions, BDI-II and finally the 20 videos of the METV test. In each part of the survey, participants were required to share their email addresses and names, which were used to link each participant’s data from each test. Data were de-identified for analyses.

### 2.5. Data screening

Participants who did not comply with the basic instructions of the study (e.g. browsers or devices that were not compatible with the METV test) were removed from the dataset. Furthermore, for all statistical analyses conducted for this study, all relevant assumptions (such as normality of data and homoscedasticity of the error terms) were verified prior to analysis.

### 2.6. Statistical analyses

For data analysis, participants were divided into one of two groups: low mood or control. The groups were created by splitting the sample into two equally sized groups based on being below or above the median BDI-II score. All data analyses were conducted using SPSS v. 28. Ordinary least squares (OLS) linear regression analyses and binary logistic regressions were conducted to test the hypotheses as follows. To test hypothesis 1 and obtain the primary outcome of the study, the initial models investigated the association between low mood and overall METV first-attempt test scores. For hypothesis 2, the dependent variable was the proportion of happy faces misjudged as neutral at first attempt. Similarly, for hypothesis 3, the dependent variable was the proportion of neutral faces misjudged as sad at first attempt. For hypothesis 4, the dependent variable was the accuracy in detecting sad faces at first attempt. In the complete models for all hypotheses, the results were adjusted for the demographic control variables age, sex and level of education. This was done to prevent possible confounding effects of these variables, as they have been found to influence facial expression recognition or cognitive performance in general in previous research [35,36]. This adjustment, alongside the comparison of the contributions of different factors was the reason for choosing the two types of regression analyses in the present study. It should be noted that the study protocol indicated that OLS linear regression would be used for all analyses. This was in error, since hypotheses 2 and 3 have binary variables as their outcomes, and binary logistic regression was used instead.

## 3. Results

### 3.1. Sample characteristics

Study data and code used for the analyses can be found on OSF.IO/6RYJ8 [37]. A total of 349 participants were included in the present study. Nine participants (2.5%) were excluded owing to missing METV scores, suggesting either problems with the presentation of videos or inattention. After excluding those with missing METV scores, the final sample size was 340 participants. There were no participants with scores outside of the 3 × interquartile range, so no outliers were excluded. Since two respondents did not report their gender, the analyses adjusted for gender as a control variable included only 338 participants. Participants’ age ranged from 19 to 81, with an average of 43.13 (s.d. = 14.40). There was an equivalent number of male and female participants (169). Only 0.3% of the participants had no formal education, 17.9% had O-levels (a subject-based qualification granted within the framework of the General Certificate of Education primarily in the UK, most commonly completed at the ages of 14–17), 29.1% had A-levels (UK subject-based qualification primarily in the UK, most commonly completed at the ages of 17 and 18), 37.9% attained a Bachelor’s degree and 14.7% had a postgraduate degree. The descriptive statistics for the main study variables may be found in table 1. Mood groups were created by splitting the sample in half based on BDI scores, with participants with scores of 12 and lower being in the control group and participants with scores of 13 and higher in the low mood group. Furthermore, histograms and descriptive statistics for the mood groups may be found in electronic supplementary material, figure S12. There were no significant differences in gender, χ^2^ (1) = 2.663, *p* = 0.103 or age, χ^2^ (4) = 5.446, *p* = 0.245, distributions between the two BDI-based groups (details in electronic supplementary material, table S1). There was a significantly higher average age in the control group (M = 45.78, s.d. = 15.20) comparedwith the low mood group (M = 40.10, s.d. = 12.73), *t* (326.574) = 3.734, *p* < 0.001. Although this difference in average age is suboptimal due to the registered effects of age on cognitive task performance [36], all analyses were controlled for age, which removes any confounding effects. The groups depicted in electronic supplementary material, figure S1, were utilized for basic analyses, while those in electronic supplementary material, figure S2, were used for exploratory analyses.

**Table 1.** Descriptive statistics of the outcome variables.

variable	M	s.d.	min	max
METV score	0.35	0.13	0.05	0.75
neutral if happy	0.04	0.20	0	1
sad if neutral	0.04	0.19	0	1
sad face METV scores	0.38	0.26	0	1

M, mean; max, maximum; METV, micro-expression training videos; min, minimum; s.d., standard deviation.

### 3.2. Relationship between low mood and micro-expression training videos test performance

Prior to interpreting the regression analyses, scatterplots of predicted against residual *Z* values were observed in order to check for assumptions, including normality and homoscedasticity of residuals. No evidence of assumption violation was found. Variance inflation factor (VIF) values close to 1 indicated no multi-collinearity.

Our results show that there is evidence for a relationship between mood and METV test scores (first correct answers) after adjusting for gender, age and education (*p* = 0.03), indicating that low mood exerts a negative effect on micro-expression recognition. When adjusting for demographics, people with low mood (participants with BDI-II scores above the median) assessed 34.1% of all facial expressions in the METV correctly on the first try. People in the control group (participants with BDI-II scores below the median) assessed 35.1% of the faces in the METV correctly on the first try.

This relationship between low mood and METV scores is not visible in the unadjusted model. However, in the adjusted model, there is evidence of a negative relationship—participants with low mood (top 50% BDI score) had worse performance on the METV. Aside from that, very strong evidence was found for an association between age and METV performance (*p* < 0.001), indicating that younger people might display higher levels of competence in recognizing human emotions from facial expressions. Full details of the models are reported in table 2.

**Table 2.** Regression models predicting the proportion of first correct answers on the micro-expression training video (METV) test.

	unadjusted		model 1	
predictors	*b* [95% CI]	*p*	*b* [95% CI]	*p*
low mood group (BDI score above median)	−0.010	0.458	−0.029	0.03
	[−0.037, 0.017]		[−0.055,−0.003]	
gender			−0.019	0.147
			[−0.044, 0.006]	
age			−0.003	< 0.001
			[−0.003,−0.003]	
education			0.006	0.65
			[−0.019, 0.031]	

Ordinary least squares regression (*n* = 338). Model 1: adjusted for age, sex and level of education. *b* = unstandardized regression coefficient, 95% CI = 95% confidence interval, *p* = probability of Type I error.

### 3.3. Relationship between low mood and perceptions of happy facial expressions

Neither unadjusted nor adjusted (i.e. age, sex and education) logistic regression analyses provided any evidence to support the hypothesis that people with low mood would perceive happy facial expressions as neutral. No evidence was found for associations between any of the demographic control variables and incorrect assessments of happy facial expressions as neutral. Full details of these models are provided in table 3.

**Table 3.** Regression models predicting misperceptions of happy facial expressions as neutral.

	unadjusted		model 1	
predictors	log odds [95% CI]	*p*	log odds [95% CI]	*p*
low mood group (BDI score above median)	−0.012	0.982	−0.061	0.913
	[−1.082, 1.058]		[−1.154, 1.033]	
age			−0.0265	0.213
			[−0.068, 0.015]	
gender			0.29941	0.59
			[−0.791, 1.389]	
education			0.79661	0.188
			[−0.391, 1.984]	

Binary logistic regression (*n* = 338). Model 1: adjusted for age, sex and level of education. 95% CI = 95% confidence interval, *p* = probability of Type I error.

### 3.4. Relationship between low mood and perceptions of neutral facial expressions

We observed a significantly positive relationship between low mood and incorrect interpretations of neutral facial expressions as sad in the adjusted (i.e. age, sex and education) logistic regression analysis (*p* = 0.042), indicating that low mood may prevent people from accurately processing neutral facial expressions. Full details of these analyses are provided in table 4. At a suggestion from one of our reviewers, we have checked whether or not the effect of misinterpreting neutral faces as sad was simply a bias towards generally answering with sad, regardless of the stimulus. As electronic supplementary material, table S5 shows, the rate of correctly identifying a stimulus as sadness and falsely interpreting any other emotion as sadness is consistent across the two groups. Also, we found no evidence of correlation between the frequency of ‘sad’ responses and misclassification of neutral faces as sad, *r* = 0.045, *p* = 0.412.

**Table 4.** Regression models predicting incorrect interpretations of neutral facial expressions as sad.

	unadjusted		model 1	
predictors	log odds [95% CI]	*p*	log odds [95% CI]	*p*
low mood group (BDI score above median)	1.235	0.064	1.414	0.042
	[−0.074, 2.543]		[0.052, 2.778]	
age			0.034	0.112
			[−0.007, 0.075]	
gender			−0.365	0.538
			[−1.527, 0.797]	
education			−0.495	0.402
			[−1.651, 0.661]	

Binary logistic regression (*n* = 338). Model 1: adjusted for age, sex and level of education. 95% CI = 95% confidence interval, *p* = probability of Type I error.

### 3.5. Relationship between low mood and perceptions of sad facial expressions

Lastly, we assessed the relationship between low mood and the recognition of sad facial expressions. Prior to interpreting the regression analyses, scatterplots of predicted against residual *Z* values were observed in order to check for assumptions, including normality and homoscedasticity of residuals. No evidence of assumption violation was found. VIF values close to 1 indicated no multi-collinearity. We found no evidence that people with higher BDI-II scores differed in their judgements of sad facial expressions from people with lower BDI-II scores.

Strong evidence was found for associations between the demographic variables and the ability to recognize sad facial expressions as measured by METV test scores. In general, older participants exhibited significantly lower METV test scores for the recognition of sad facial expressions (*p* < 0.001). Additionally, male participants exhibited significantly worse sad face METV test scores than female participants (*p* = 0.004). Lastly, participants with a higher level of education exhibited significantly worse sad face METV test scores (*p* = 0.044). Full details of these models are provided in table 5.

**Table 5.** Regression models predicting the recognition of sad facial expressions.

	unadjusted		model 1	
predictors	*b* [95% CI]	*p*	*b* [95% CI]	*p*
low mood group (BDI score above median)	0.009	0.99	−0.959	0.171
	[−1.388, 1.405]		[−2.335, 0.417]	
age			−0.119	< 0.001
			[-0.166,-0.072]	
gender			−1.988	0.004
			[-3.331,-0.645]	
education			−1.387	0.044
			[-2.734,-0.04]	

Ordinary least squares regression (*n* = 338). Model 1: adjusted for age, sex and level of education. *b* = unstandardized regression coefficient, 95% CI = 95% confidence interval, *p* = probability of Type I error.

### 3.6. Assessment of least and most depressed quartiles

An exploratory analysis of the extreme mood quartiles of participants (based on BDI-II scores) provided some evidence for the wider notion that people with low mood have more difficulty with correctly perceiving happy facial expressions. In this analysis, we observed a significant negative association between BDI-II scores and happy expression recognition (first correct answers) when the analysis was adjusted for gender, age and education (*p* = 0.03). The exponential regression coefficient of exp(*B*) = 0.448 indicates that the odds of the high BDI scores (top 25%) group getting the happy face correctly are less than half (44.8%) of the same odds in the low BDI scores group (bottom 25%). In other words, participants in the low BDI scores group were more likely to correctly identify the happiness micro-expression in comparison with the participants in the high BDI scores group. Full details of these models are provided in table 6. There were no effects of the extreme mood groups variable on overall METV scores, recognizing neutral faces as sad or recognizing sad faces (electronic supplementary material, tables S2–S4).

**Table 6.** Regression models predicting the correct recognition of happy facial expressions on the METV test.

	unadjusted		model 1	
predictors	log odds [95% CI]	*p*	log odds [95% CI]	*p*
very low mood group (top 25% BDI score)	0.509	0.046	0.448	0.033
	[0.262, 0.987]		[0.214, 0.936]	
age			0.991	0.478
			[0.966, 1.017]	
gender			1.332	0.401
			[0.682, 2.601]	
education			0.642	0.198
			[0.327, 1.260]	

Binary logistic regression (*n* = 178). Model 1: adjusted for age, sex and level of education. 95% CI = 95% confidence interval, *p* = probability of Type I error.

### 3.7. Sensitivity analysis

In order to determine whether or not antidepressant usage by a portion of the sample affected study findings, sensitivity analysis was performed by excluding these participants from the sample and re-doing the main study analysis. The point estimate of the effect of mood group on METV score in the adjusted model was closer to zero, and there was no evidence of an effect (electronic supplementary material, table S6). The effect of age remained the same. Therefore, excluding participants who used antidepressants only reduced the statistical power of the study, without changing the direction of any of the effects.

## 4. Discussion

The present study is the first to assess the relationship between low mood and the recognition of micro-facial expressions, as assessed by METV test scores. We found that low mood can significantly affect the perception of facial micro expressions, indicating that people with low mood may have a more difficult time discerning micro-expressions of emotions during interpersonal interactions. This is in agreement with several previous studies that investigated the impact of depression on emotion recognition abilities [29,38–40]. Two meta-analytic studies [11,41] showed consistent evidence supporting impaired emotion recognition in individuals with depression. Other studies involving facial expressions, vocal intonations and other non-verbal cues mostly affirm that people with depression fare worse than the average individual at recognizing emotions [39]. We also found that there was no robust association between BDI-II scores and the misperception of happy facial expressions as neutral. Our analysis of the groups of participants with lowest and highest BDI scores provided evidence for the wider notion that people with lower mood have more difficulty with correctly assessing happy facial expressions. It should be noted that each participant only saw one happy micro-expression during the study, which may have limited the possibility of detecting differences between groups. Although previous research found associations between symptoms of depression and emotion recognition, this study has been the first to do so using videos of micro-expressions, which have higher ecological validity when it comes to micro-expression detection. The fact that the findings are mostly in agreement with previous literature indicates that the processes involved in detecting micro-expressions and macro-expressions might be similar and reduced in a similar way due to depressive symptomatology.

Depression is a heterogeneous disorder, and individuals living with depression can experience a wide range of symptom severity [42], including the intensity and duration of depressive symptoms. Individuals with mild symptoms of depression may possess the ability to accurately identify emotions [43], as observed in the current study with emotion recognition abilities, with deficits only visible in subgroup analyses of extreme cases. On the other hand, severely depressed individuals may exhibit impairments in emotion recognition owing to the phenomenon of lower cognitive ability associated with depression thought to affect all mental functions—even daily activities such as eating, bathing and mobility [44]. The present study was not able to adequately investigate that due to a low number of participants with high scores on the BDI-II.

In addition, we observed an association between low mood and the propensity of the participants to misjudge neutral faces as sad. This finding can be understood through the lens of mood congruence, a phenomenon in which an individual’s current emotional state influences the processing and recall of information that is congruent with that mood [10]. In people with depression, negative mood congruence may lead individuals to perceive neutral stimuli through a negative emotional lens, attributing sadness to otherwise neutral facial expressions [10]. In addition, depression is associated with negative cognitive biases, wherein individuals are more likely to attend to, recall and interpret negative information [45]. This negative bias extends to facial expressions, leading individuals with depression to focus more on negative emotional cues in facial expressions than on neutral or positive ones. Consequently, neutral facial expressions may be mistakenly perceived as sad due to heightened attention to negative emotional cues [46]. Aside from attentional bias, it is also important to have in mind the interpretation bias. Findings from the literature indicate that more depressed individuals are more likely to interpret ambiguous stimuli as negative [10]. This can lead to other difficulties in their everyday life. For instance, they might experience events in their social relationship that would be neutral for others but negative for them. This would leave them further convinced of negative occurrences in their lives and may further validate their feelings of depression. That is why working on cognitive biases, such as the interpretation bias, is a cornerstone of cognitive behavioural therapy for depression [47].

Older participants exhibited worse recognition of sad facial expressions. Previous research has also found that there are declines in specific emotion recognition [36,48,49] with age, which is explained by a decline in the volume of the ‘social brain’, mainly in the frontal and temporal lobes [50]. Additionally, in the current study, male participants exhibited significantly worse sad face METV test scores than female participants. This is consistent with past findings in the fields of evolutionary psychology and neurobiology and is attributed to differences in specific patterns of neural responses to emotional faces [35,51–53]. Lastly, participants with a higher level of education exhibited significantly worse sad face METV test scores. While this may seem counterintuitive, this is also in line with findings of previous research [54].

The study’s findings underscore the challenges that people with symptoms of depression face in perceiving and understanding micro-facial expressions during interpersonal interactions. Depression already creates a barrier to effective communication and social engagement. The impaired recognition of emotions through facial cues intensifies this challenge. Such difficulties might contribute to the perception of social isolation and exacerbate feelings of alienation, as individuals with depression may struggle to connect with others on an emotional level. This, in turn, can perpetuate a cycle of negative emotions and reinforce depressive symptoms.

Furthermore, the study highlights how the perception of neutral facial expressions as sad among individuals with depression could be attributed to their negative cognitive biases and mood congruence. These biases could distort their understanding of others’ emotions, leading them to perceive more negativity in social interactions than intended. This misinterpretation might lead to misunderstandings, miscommunication and a heightened sense of social inadequacy.

### 4.1. Limitations

The BDI tool used to measure depression symptoms (which were interpreted as mood) and the METV tool to measure the ability to recognize sad faces might not have been sensitive enough to capture subtle differences between groups [55]. Furthermore, owing to the fact that there was only one happy and one neutral face presented to the participants, there may not have been enough variation among the participants on these variables to detect some of the investigated effects. This may have also introduced some bias in the sensitivity of detecting different facial expressions in the task. However, since there is only one variation in facial muscle movement representing happiness—lip corners raising—having multiple instances of it could potentially lead to a learning effect. For all other emotions that have more variations of facial movements, participants were exposed to only one representation of each movement. Therefore, incorporating multiple instances of the same movement could introduce another form of bias. Ultimately, a compromise was necessary, but it would be advisable for future studies to assess whether the effects observed in the current study persist with an alternative approach.

The present research did not utilize recommended common measures in mental health research [56], which reduces its comparability with other mental health studies. Furthermore, the faces used in the present study were of white people, and we did not record the ethnicity/race of participants, which means that, were there any effects of these variables on the study results, they could not be measured.

Antidepressant usage was not taken into account in the present study, since there was an insufficiently small number of participants who reported its usage for analysis, but large enough for the statistical power of the study to be reduced, were they removed. Therefore, they were considered a part of the sample. Similarly, we did not monitor for past depressive episodes. While research [18] has demonstrated that there may be a latent effect of depression on facial expression recognition even after remission, this phenomenon may also occur in individuals who are not currently experiencing depression. Therefore, it was not deemed relevant for the present study. Future research may want to differentiate the results based on antidepressant usage. Finally, while the effects of other mental health issues, such as anxiety, on micro-expression recognition are also recognized in the literature [11], these effects were not taken into account in the present study.

In sum, future research may aim to use the present study as a basis and build upon it by controlling more factors (anxiety, ethnicity of stimuli and participants, and depression history), using measures proposed by the International Alliance of Mental Health Research Funders (IAMHRF) [56] and using more stimuli representing the same expressions. These improvements could help improve the generalizability and determine the robustness of the present study findings.

## 5. Conclusions

This study assessed the relationship between the recognition of micro-facial expressions and low mood. The results of the study show an association between low mood and performance on the METV test, providing yet another finding supporting the known effects of depression on various aspects of our social functioning. This study found no evidence of an association between depression and misjudging happy facial expressions as neutral ones. However, the fact that there was only one happy and one neutral face presented to the participants may have led of a lack in variation among the participants needed to detect these hypothesized effects. Deeper analyses of the least depressed and most depressed participants revealed that the people who were most depressed experienced difficulty with correctly assessing happy facial expressions. Furthermore, we found that people with depression apply a negative bias to neutral micro-expressions. These findings suggest that people with depression might experience difficulties in accurately detecting emotions in encounters with other people and may have an inherent negative bias when interpreting facial expressions in conversation. This work begins to elucidate the relationship between micro-expression recognition and depression, with broader implications for how misperceptions of micro-expressions could affect interpersonal interactions, relationships and quality of life. Moreover, it can help facilitate the development of interventions to mitigate the social effects of depression. Future work will use the METV test to further clarify the relationship between mental health disorders and micro-expression recognition, and ultimately to design interventions that could help mitigate the effects of these disorders on social life and quality of life.

## Data Availability

Study data and analysis code is available on OSF [37]. Supplementary material is available online [57].

## References

[1] Drevets WC , Todd RD . 2005 Depression, mania, and related disorders. In Adult psychiatry (eds EH Rubin , CF Zorumski ), pp. 91–129, 2nd edn. Malden, MA: Blackwell Publishing.

[2] Rottenberg J , Kasch KL , Gross JJ , Gotlib IH . 2002 Sadness and amusement reactivity differentially predict concurrent and prospective functioning in major depressive disorder. Emotion **2** , 135–146. (10.1037/1528-3542.2.2.135) 12899187

[3] Shorey S , Ng ED , Wong CHJ . 2022 Global prevalence of depression and elevated depressive symptoms among adolescents: a systematic review and meta-analysis. Br. J. Clin. Psychol. **61** , 287–305. (10.1111/bjc.12333) 34569066

[4] Fernández-Theoduloz G , Paz V , Nicolaisen-Sobesky E , Pérez A , Buunk AP , Cabana Á , Gradin VB . 2019 Social avoidance in depression: a study using a social decision-making task. J. Abnorm. Psychol. **128** , 234–244. (10.1037/abn0000415) 30920233

[5] Kurimoto P , Lueboonthavatchai P , Maes M . 2020 Impaired social skills as a key component of clinical depression: associations with severity of illness, self-esteem, family functional health satisfaction, and personality features. Preprints. (10.20944/preprints202005.0122.v1)

[6] Persad SM , Polivy J . 1993 Differences between depressed and nondepressed individuals in the recognition of and response to facial emotional cues. J. Abnorm. Psychol. **102** , 358–368. (10.1037//0021-843x.102.3.358) 8408947

[7] Suffel A , Nagels A , Steines M , Kircher T , Straube B . 2020 Feeling addressed! The neural processing of social communicative cues in patients with major depression. Hum. Brain Mapp. **41** , 3541–3554. (10.1002/hbm.25027) 32432387 PMC7416026

[8] Nejati V . 2018 Negative interpretation of social cue in depression: evidence from reading mind from eyes test. Neurol. Psychiatry Brain Res. **27** , 12–16. (10.1016/j.npbr.2017.11.001)

[9] Everaert J , Podina IR , Koster EHW . 2017 A comprehensive meta-analysis of interpretation biases in depression. Clin. Psychol. Rev. **58** , 33–48. (10.1016/j.cpr.2017.09.005) 28974339

[10] LeMoult J , Gotlib IH . 2019 Depression: a cognitive perspective. Clin. Psychol. Rev. **69** , 51–66. (10.1016/j.cpr.2018.06.008) 29961601 PMC11884012

[11] Demenescu LR , Kortekaas R , den Boer JA , Aleman A . 2010 Impaired attribution of emotion to facial expressions in anxiety and major depression. PLoS One **5** , e15058. (10.1371/journal.pone.0015058) 21152015 PMC2995734

[12] Ruihua M *et al* . 2021 Differences in facial expression recognition between unipolar and bipolar depression. Front. Psychol. **12** , 619368. (10.3389/fpsyg.2021.619368) 34335353 PMC8316620

[13] Zhu C , Chen X , Zhang J , Liu Z , Tang Z , Xu Y , Zhang D , Liu D . 2017 Comparison of ecological micro-expression recognition in patients with depression and healthy individuals. Front. Behav. Neurosci. **11** , 199. (10.3389/fnbeh.2017.00199) 29089879 PMC5651037

[14] Zhu C , Yin M , Chen X , Zhang J , Liu D . 2019 Ecological micro-expression recognition characteristics of young adults with subthreshold depression. PLoS One **14** , e0216334. (10.1371/journal.pone.0216334) 31042784 PMC6493753

[15] Baruch N , Behrman S , Wilkinson P , Bajorek T , Murphy SE , Browning M . 2021 Negative bias in interpretation and facial expression recognition in late life depression: a case control study. Int. J. Geriatr. Psychiatry **36** , 1450–1459. (10.1002/gps.5557) 33900662

[16] Douglas KM , Porter RJ . 2010 Recognition of disgusted facial expressions in severe depression. Br. J. Psychiatry **197** , 156–157. (10.1192/bjp.bp.110.078113) 20679270

[17] Porter-Vignola E , Booij L , Bossé-Chartier G , Garel P , Herba CM . 2021 Emotional facial expression recognition and depression in adolescent girls: associations with clinical features. Psychiatry Res. **298** , 113777. (10.1016/j.psychres.2021.113777) 33581380

[18] Milders M , Bell S , Platt J , Serrano R , Runcie O . 2010 Stable expression recognition abnormalities in unipolar depression. Psychiatry Res. **179** , 38–42. (10.1016/j.psychres.2009.05.015) 20478626

[19] Bhagwagar Z , Cowen PJ , Goodwin GM , Harmer CJ . 2004 Normalization of enhanced fear recognition by acute SSRI treatment in subjects with a previous history of depression. Am. J. Psychiatry **161** , 166–168. (10.1176/appi.ajp.161.1.166) 14702268

[20] Frank MG , Maccario CJ , Govindaraju V . 2009 Behavior and security. In Protecting airline passengers in the age of terrorism (eds P Seidenstat , FX Splane ), pp. 86–106. Santa Barbara, CA: Greenwood Pub Group. (10.5040/9798216002246)

[21] Matsumoto D . 2001 Culture and emotion. In The handbook of culture and psychology (ed. D Matsumoto ), pp. 171–194. New York, NY: Oxford University Press.

[22] Matsumoto D , Keltner D , Shiota M , Frank M , O’Sullivan M . 2008 What’s in a face? Facial expressions as signals of discrete emotions. In Handbook of emotions (eds M Lewis , JM Haviland , LF Barrett ), pp. 211–234. New York, NY: Guilford Press.

[23] Matsumoto D , Hwang HS . 2011 Evidence for training the ability to read microexpressions of emotion. Motiv. Emot. **35** , 181–191. (10.1007/s11031-011-9212-2)

[24] Ekman P . 2003 Emotions revealed: recognizing faces and feelings to improve communication and emotional life, 1st edn. New York, NY: Times Books.

[25] Hurley CM . 2012 Do you see what I see? Learning to detect micro expressions of emotion. Motiv. Emot. **36** , 371–381. (10.1007/s11031-011-9257-2)

[26] Qu F , Wang SJ , Yan WJ , Fu X . 2016 CAS(ME)^2^: a database of spontaneous macro-expressions and micro-expressions. In Human-computer interaction. novel user experiences. HCI 2016 (ed. M Kurosu ), pp. 48–59. Cham, Switzerland: Springer. (10.1007/978-3-319-39513-5_5)

[27] Beck AT , Steer RA , Brown GK . 1996 Manual for the beck depression inventory-II. San Antonio, TX: Psychological Corporation.

[28] Wezowski K , Maison D . 2016 Reading facial expression to understand human emotions: micro-expressions training videos (METV): the new tool for experimental economics. In Selected issues in experimental economics (eds K Nermend , M Łatuszyńska ), pp. 135–149. Cham, Switzerland: Springer International Publishing. (10.1007/978-3-319-28419-4_10)

[29] Dalili MN , Penton-Voak IS , Harmer CJ , Munafò MR . 2015 Meta-analysis of emotion recognition deficits in major depressive disorder. Psychol. Med. **45** , 1135–1144. (10.1017/S0033291714002591) 25395075 PMC4712476

[30] Faul F , Erdfelder E , Lang AG , Buchner A . 2007 G*Power 3: a flexible statistical power analysis program for the social, behavioral, and biomedical sciences. Behav. Res. Methods **39** , 175–191. (10.3758/bf03193146) 17695343

[31] Wang YP , Gorenstein C . 2013 Psychometric properties of the Beck Depression Inventory-II: a comprehensive review. Braz. J. Psychiatry **35** , 416–431. (10.1590/1516-4446-2012-1048) 24402217

[32] Ekman P , Friesen WV . 1978 Facial Action Coding System (FACS). APA PsycTests. (10.1037/t27734-000)

[33] Yu EH , Choi EJ , Lee SY , Im SJ , Yune SJ , Baek SY . 2016 Effects of micro- and subtle-expression reading skill training in medical students: a randomized trial. Patient Educ. Couns. **99** , 1670–1675. (10.1016/j.pec.2016.04.013) 27134051

[34] Wezowski K , Wezowski P . 2012 The micro expressions book for business: how to read facial expressions for more effective negotiations, sales and recruitment, 1st edn. Kampala, Uganda: New Vision.

[35] Lee TMC , Liu HL , Hoosain R , Liao WT , Wu CT , Yuen KSL , Chan CCH , Fox PT , Gao JH . 2002 Gender differences in neural correlates of recognition of happy and sad faces in humans assessed by functional magnetic resonance imaging. Neurosci. Lett. **333** , 13–16. (10.1016/s0304-3940(02)00965-5) 12401549

[36] Seblova D , Berggren R , Lövdén M . 2020 Education and age-related decline in cognitive performance: systematic review and meta-analysis of longitudinal cohort studies. Ageing Res. Rev. **58** , 101005. (10.1016/j.arr.2019.101005) 31881366

[37] Wezowski K , Penton-Voak I . 2024 Association between depression and micro expression processing (dataset) OSF. (10.17605/OSF.IO/6RYJ8) 37804702

[38] Anderson IM *et al* . 2011 State-dependent alteration in face emotion recognition in depression. Br. J. Psychiatry **198** , 302–308. (10.1192/bjp.bp.110.078139) 21263011

[39] Joormann J , Gotlib IH . 2006 Is this happiness I see? Biases in the identification of emotional facial expressions in depression and social phobia. J. Abnorm. Psychol. **115** , 705–714. (10.1037/0021-843X.115.4.705) 17100528

[40] Krause FC , Linardatos E , Fresco DM , Moore MT . 2021 Facial emotion recognition in major depressive disorder: a meta-analytic review. J. Affect. Disord. **293** , 320–328. (10.1016/j.jad.2021.06.053) 34229285 PMC8457509

[41] Bora E , Berk M . 2016 Theory of mind in major depressive disorder: a meta-analysis. J. Affect. Disord. **191** , 49–55. (10.1016/j.jad.2015.11.023) 26655114

[42] Olvet DM , Klein DN , Hajcak G . 2010 Depression symptom severity and error-related brain activity. Psychiatry Res. **179** , 30–37. (10.1016/j.psychres.2010.06.008) 20630603

[43] Li M , Feng L , Liu X , Zhang M , Fu B , Wang G , Lu S , Zhong N , Hu B . 2018 Emotional working memory in patients with major depressive disorder. J. Int. Med. Res. **46** , 1734–1746. (10.1177/0300060518758225) 29529905 PMC5991227

[44] McCall WV , Dunn AG . 2003 Cognitive deficits are associated with functional impairment in severely depressed patients. Psychiatry Res. **121** , 179–184. (10.1016/j.psychres.2003.09.003) 14656452

[45] Reinen JM , Whitton AE , Pizzagalli DA , Slifstein M , Abi-Dargham A , McGrath PJ , Iosifescu DV , Schneier FR . 2021 Differential reinforcement learning responses to positive and negative information in unmedicated individuals with depression. Eur. Neuropsychopharmacol. **53** , 89–100. (10.1016/j.euroneuro.2021.08.002) 34517334 PMC8633147

[46] Van Vleet T , Stark-Inbar A , Merzenich MM , Jordan JT , Wallace DL , Lee MB , Dawes HE , Chang EF , Nahum M . 2019 Biases in processing of mood-congruent facial expressions in depression. Psychiatry Res. **275** , 143–148. (10.1016/j.psychres.2019.02.076) 30908978 PMC6504610

[47] Beck, M.D. AT , Alford, Ph.D. BA . 2009 Depression. In Causes and treatment, 2nd edn. Philadelphia, PA: University of Pennsylvania Press. (10.9783/9780812290882). See https://www.degruyter.com/document/doi/10.9783/9780812290882/html.

[48] Tucker-Drob EM , Brandmaier AM , Lindenberger U . 2019 Coupled cognitive changes in adulthood: a meta-analysis. Psychol. Bull. **145** , 273–301. (10.1037/bul0000179) 30676035 PMC6375773

[49] Lövdén M , Fratiglioni L , Glymour MM , Lindenberger U , Tucker-Drob EM . 2020 Education and cognitive functioning across the life span. Psychol. Sci. Public Interest **21** , 6–41. (10.1177/1529100620920576) 32772803 PMC7425377

[50] Ruffman T , Henry JD , Livingstone V , Phillips LH . 2008 A meta-analytic review of emotion recognition and aging: implications for neuropsychological models of aging. Neurosci. Biobehav. Rev. **32** , 863–881. (10.1016/j.neubiorev.2008.01.001) 18276008

[51] Connolly HL , Lefevre CE , Young AW , Lewis GJ . 2019 Sex differences in emotion recognition: evidence for a small overall female superiority on facial disgust. Emotion **19** , 455–464. (10.1037/emo0000446) 29781645

[52] McClure EB . 2000 A meta-analytic review of sex differences in facial expression processing and their development in infants, children, and adolescents. Psychol. Bull. **126** , 424–453. (10.1037/0033-2909.126.3.424) 10825784

[53] Dodich A , Cerami C , Canessa N , Crespi C , Marcone A , Arpone M , Realmuto S , Cappa SF . 2014 Emotion recognition from facial expressions: a normative study of the Ekman 60-faces test in the Italian population. Neurol. Sci. **35** , 1015–1021. (10.1007/s10072-014-1631-x) 24442557

[54] Trauffer NM , Widen SC , Russell JA . 2013 Education and the attribution of emotion to facial expressions. Psih. teme **22** , 942–957.

[55] Schneibel R , Brakemeier EL , Wilbertz G , Dykierek P , Zobel I , Schramm E . 2012 Sensitivity to detect change and the correlation of clinical factors with the Hamilton Depression Rating Scale and the Beck Depression Inventory in depressed inpatients. Psychiatry Res. **198** , 62–67. (10.1016/j.psychres.2011.11.014) 22445070

[56] IAMHRF . 2020 Driving the adoption of common measures. See https://iamhrf.org/projects/driving-adoption-common-measures#faqs.

[57] Wezowski K , Penton-Voak I . 2024 Data from: Relationship between low mood and micro expression processing: evidence of negative bias in interpreting fleeting facial expressions. Figshare. (10.6084/m9.figshare.c.7362259) PMC11288663 39086818

